# Impact of supermarket fruit and vegetable placement on store sales, customer purchasing, diet and household waste: A prospective matched-controlled cluster trial

**DOI:** 10.1371/journal.pmed.1004575

**Published:** 2026-03-31

**Authors:** Christina Vogel, Sarah Crozier, Preeti Dhuria, Joanne Lord, Graham Moon, Wendy Lawrence, Janet Cade, Kylie Ball, Cyrus Cooper, Janis Baird

**Affiliations:** 1 Centre for Food Policy, City St George’s, University of London, London, United Kingdom; 2 Medical Research Council Lifecourse Epidemiology Centre, University of Southampton, Southampton General Hospital, Southampton, United Kingdom; 3 NIHR Applied Research Collaboration Wessex, Southampton Science Park, Southampton, United Kingdom; 4 National Institute for Health Research Southampton Biomedical Research Centre, University of Southampton and University Hospital Southampton NHS Foundation Trust, Southampton, United Kingdom; 5 Southampton Health Technology Assessments Centre, Wessex Institute, University of Southampton, Alpha House, Southampton, United Kingdom; 6 Geography and Environmental Science, University of Southampton, Southampton, United Kingdom; 7 Primary Care, Population Science and Medical Education, Faculty of Medicine, University of Southampton, University Road, Southampton, United Kingdom; 8 School of Food Science and Nutrition, University of Leeds, Leeds, United Kingdom; 9 Institute of Physical Activity and Nutrition Research, School of Exercise and Nutrition Sciences, Deakin University, Geelong, Victoria, Australia; Carolina Population Center, UNITED STATES OF AMERICA

## Abstract

**Background:**

Previous product placement trials have been underpowered and limited in outcomes. This study assessed effects of positioning an expanded fruit and vegetable section near entrances on store-level sales, household-level purchasing and waste, and dietary behaviours.

**Methods and findings:**

This prospective matched controlled cluster trial (NIHR 17/44/46) involved 36 stores (18 intervention and 18 control) of a discount supermarket chain in England. The study took place between March 2018 and May 2022, and the intervention was implemented for six months. Control stores were matched on store sales, customer profiles and neighbourhood deprivation. Women customers aged 18–60 years, with loyalty cards, who shopped at intervention (*n* = 280) or control (*n* = 300) stores agreed to participate. The primary outcome was household purchasing of fresh fruit and vegetables. Secondary outcomes included: i) differences in household purchasing by educational attainment, ii) store sales of fresh fruit and vegetables, iii) dietary quality score for woman, and iv) child aged 2−6 years (if relevant), and v) household fruit and vegetable waste. The proportion of households purchasing fruit and vegetables in intervention compared to control stores was very similar at baseline (−0.1% (95%CI −6.1%, 6.0%)). After 3 months of exposure to the intervention, the proportion was 0.6% (95%CI −5.6%, 6.7%; *p =* 0.83) and after 6 months the proportion was 3.3% (95%CI −2.5%, 9.2%; *p =* 0.23). Interrupted time series analyses showed differences in intervention compared to predicted store-level sales of fruit and vegetables were 0.32SDs (95%CI 0.11, 0.53; *p* = 0.002) at intervention implementation, equivalent to ~2,525 (95% CI 775, 4,115) extra portions per store, per week. The differences were 0.23SDs (95%CI −0.05, 0.52; *p* = 0.10) at 3 months and 0.18SDs (−0.16, 0.52); *p* = 0.29) at 6 months post-intervention. Not being able to randomise stores potentially biases the results through unmeasured confounding effects and findings related to intervention dose were not prespecified but determined from process evaluation findings investigating intervention implementation.

**Conclusions:**

This study was conducted during the COVID-19 pandemic and cost-of-living crisis when population level fruit and vegetable sales and intake declined and recruitment to research was challenging. Despite these circumstances, the results of this study show that positioning produce sections near supermarket entrances may improve the nutrition profile of store sales, household purchasing and women’s dietary quality.

**Trial registration:**

NCT03573973.

## Author summary

**Why was this study done?:**

- Obesity and poor diet are major public health concerns and retailers’ marketing strategies impact food choices
- Product placement is a marketing strategy used by many retailers to promote unhealthy foods
- Robustly designed real-world studies on the prominent placement of healthy foods can inform improvements to existing regulations to maximise their impact on population diet

**What did the researchers do and find?:**

- We evaluated a natural experiment that was implemented across a UK discount supermarket chain to assess whether moving the fresh fruit and vegetable section to near store entrances and expanding the range available changes store sales, household purchasing and individual diets
- Despite substantial declines in population fruit and vegetable purchasing and intake over the study period, positioning an expanded fruit and vegetable section near store entrances increased fresh fruit and vegetable sales at the population level by approximately 2,525 extra portions per store, per week

**What do these findings mean?:**

- Government regulations to curb placement marketing strategies being used by retailers to promote unhealthy foods should consider requiring the placement of a fruit and vegetables section at store entrances alongside limiting placement of unhealthy foods in locations such as checkouts, aisle-ends and store entrances to maximise their health benefit
- This intervention may protect against declines in household produce purchasing, particularly for those exposed to a greater intervention dose or experiencing greater socioeconomic deprivation although these findings were not statistically robust
- Not being able to randomise stores potentially biases the results through unmeasured confounding effects and findings related to intervention dose were not prespecified but determined from process evaluation findings investigating intervention implementation

## Abstract 3

Christina Vogel and colleagues assessed the impact of moving the fresh fruit and vegetable section near store entrances on store-level sales, household-level purchasing and waste, and dietary behaviours.

## 1. Introduction

Obesity and poor diet constitute two of the greatest threats to population health. They are key priorities of government policies because they contribute directly to poor productivity, mortality and health inequalities [1]. There are widening inequalities in diet, weight status and life expectancy between socioeconomically vulnerable and affluent families [2].

The food industry and citizens are trapped in a ‘*junk food cycle’*, where unhealthy foods are cheap to make, profitable to market, appealing to eat and affordable to buy [3]. Healthy food is twice as expensive per calorie than unhealthy foods high in fat, sugar and salt (HFSS), and <1% of placement promotions are for fruit and vegetables [4,5]. In recognition of the need for policies that help shape food environments to support everyone to achieve and sustain a healthy diet, the United Kingdon (UK) Government implemented the first component of the Food (Promotions and Placement) regulations on 1st October 2022 in England [6]. This law supports the creation of healthier store layouts in all large retailers by restricting the positioning of HFSS products in prominent in-store locations including store entrances, aisle-ends and checkouts. Pre-implementation assessment of these regulations indicated widespread support for these new rules, however, some stakeholders felt the restrictions could go further in supporting everyone to make healthier food choices [7–10].

Reviews of the scientific literature assessing the effectiveness of product placement strategies indicate moderate evidence for healthier positioning strategies improving diet-related behaviours such as store sales, household purchasing and dietary patterns. Effects are stronger when healthy foods are positioned in prominent locations and unhealthy foods are concurrently positioned in less prominent locations, and there is greater consistency of effects when healthier positioning strategies are combined with increasing availability of healthy foods or reducing availability of unhealthy products [11,12]. Few studies in this field of research are well designed due to methodological difficulties implementing robust study designs in real-world retail settings [11,13]. There is also limited evidence of the differential effects by socioeconomic status, yet this information is particularly important for policy makers [13,14].

While many supermarkets do place fresh fruit and vegetables in a position that customers encounter when first entering the store, discount and small supermarket chains do not routinely place fruit and vegetables near the store entrance. UK research shows that discount and small supermarkets have less healthy environments than other UK supermarkets, including lower availability and less prominent placement of fresh fruit and vegetables [15,16]. These poorer in-store environments may be contributing to dietary inequalities because families experiencing disadvantage and younger adults, known to have poorer quality diets, frequently rely on these stores for their food [17,18]. Evaluation of the effects of this placement strategy could inform future improvements to existing government regulations.

This study addresses several evidence gaps regarding the use of placement strategies to improve population diet. It aims to assess whether enhancing the positioning and availability of the fresh produce section in discount supermarkets in England improves the healthiness of store sales (population level, secondary outcome), household purchasing (household level, primary outcome), and dietary quality among women customers aged 18–60 years and their young children (individual level, secondary outcomes) after 3 and 6 months. To our knowledge, this study is unique in its analysis of individual loyalty card data, alongside store sales data, and dietary data from more than one family member [19,20]. The study also assessed household fruit and vegetables waste patterns to provide a wholistic evaluation.

## 2. Methods

### 2.1. Study design and setting

**Fig 1:**
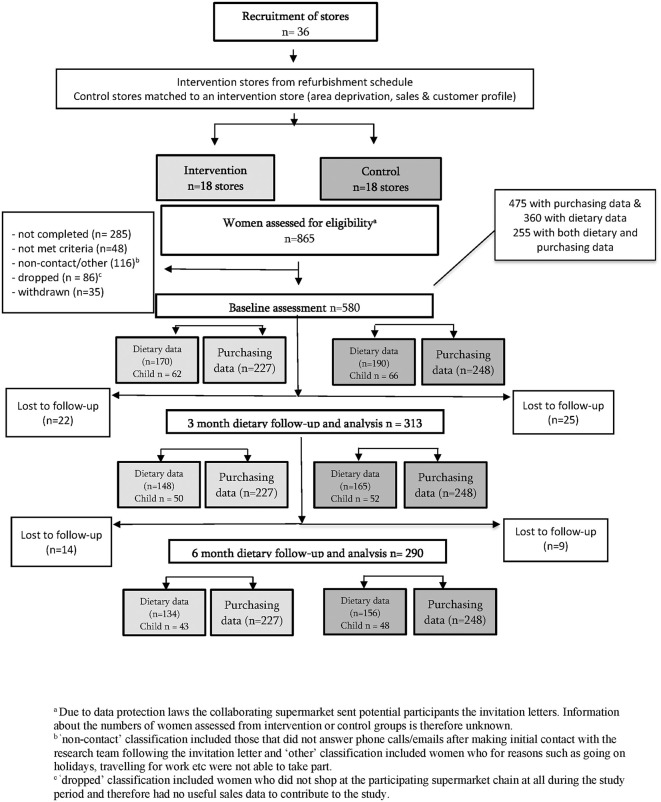
WRAPPED study flow chart and sample size.

The WRAPPED study was a natural experiment with a prospective matched controlled cluster design, with participants clustered within 36 study supermarkets to account for the store-based intervention. The flow diagram, Fig 1, illustrates the sampling frame for all outcome data points: store sales, customer purchasing, dietary data and household waste. The study took place between March 2018 and May 2022 and was registered with ClinicalTrials.gov (prospectively registered NCT03573973). It was approved by the University of Southampton, Faculty of Medicine Ethics Committee (ID 20986.A9) and conducted in accordance with the Declaration of Helsinki and Data Protection regulations. Modifications from the original protocol [21] were approved by both the University of Southampton, Faculty of Medicine Ethics Committee and the independent Study Steering Committee. The updated protocol is available on the project funder’s website [22]. This study is reported as per Consolidated Standards of Reporting Trials (CONSORT and CONSERVE) guidelines (S1 CONSORT Checklist and S1 CONSERVE Checklist).

The study setting was discount supermarket stores in England. The collaborating supermarket has over 1,000 stores nationwide and holds ~2% of the UK grocery market share [23].

This study sampled 36 stores, 18 intervention and 18 control stores [21]. Intervention stores were selected by the research team from the full list of stores due for refurbishment and structural changes to their in-store environment during the study period by the company. Retail store refurbishment occurs for all stores roughly every 5–7 years to keep stores’ decor fresh and equipment energy efficient. Intervention stores were selected by the research team from company planograms. Any store set for refurbishment which did not have the fruit and vegetable section in the first aisle previously were included. Control stores were matched to an intervention store on: i) sales profile, ii) customer profile, and iii) neighbourhood deprivation (Index of Multiple Deprivation (IMD)) [13,24]. Control stores were geographically distant from intervention stores to reduce contamination effects.

### 2.2. Intervention and control conditions

The intervention was implemented continuously for 6 months with two components executed simultaneously: i) more prominent positioning of fresh fruit and vegetables near store entrances, and ii) expanding the numbers of fresh fruit and vegetables available. The positioning or availability of frozen and canned fruit and vegetables were not altered.

The control condition was the previous layout of stores with a limited range of fresh fruit and vegetables, placed at the back of the store and other products in their usual locations.

### 2.3. Participant eligibility and recruitment

Women aged 18–60 years were the target population because improving the diets of women in this age group will improve their own health, and the short and long-term health of their children [25,26]. Women within this age group are also primarily responsible for domestic food-related tasks that influence their partners and families’ diets [27,28]. Eligible participants were women aged 18–60 years, holding a study supermarket loyalty card and had shopped in a study store 12 weeks before recruitment. Women under 18 or over 60 years of age, without a loyalty card or who shopped online only were not eligible.

Recruitment occurred in five waves between May 2018 and October 2021 with each pair of stores recruited over the same pre-implementation period. Eligible women, identified from loyalty card data, were sent a letter inviting them to participate. Participation involved completing four telephone interviews with a researcher at baseline and 1, 3 and 6 months after intervention implementation, and providing oral consent to be interviewed and written consent to share their loyalty card data. The letter did not contain details about the intervention and was sent by the supermarket to comply with data protection laws. Interested women contacted the research team directly via Freephone, text or email, were screened for eligibility and provided informed consent. To boost participant numbers, in-store recruitment was employed. The research team approached women customers while shopping in study stores and offered an information sheet. Interested women were subsequently phoned and consented. This method proved effective at enhancing sample diversity and was used for all 36 stores. Participants were offered up to 3× £10 Love2Shop or Amazon vouchers for their time. Each £10 voucher was sent to participants after completion of the baseline, 3- and 6-month questionnaires.

Due to COVID-19 restrictions, in-store recruitment was not possible and fewer participants responded to invitation letters than had done in the study’s pilot phase [21,24]. Consequently, the age criteria for participants were expanded from 18–45 years to 18–60 years and a sixth wave of recruitment was undertaken between September 2022 and February 2023 to boost purchasing data numbers (primary outcome) according to post-hoc sample size calculations (S1 Box). A letter was sent by the collaborating supermarket chain. Participants were offered a £15 Love2Shop or Amazon voucher for completing one telephone interview and consenting to share their loyalty card data.

All participants completed a demographic questionnaire about their age, ethnicity, marital status, highest educational qualification, employment status, number of children, weekly money spent on groceries and whether they shopped for most of their household’s food from the study supermarket chain. Participants provided home postcodes to identify neighbourhood deprivation status (IMD).

### 2.4. Outcome measures

Data collected included 9 months continuous *store sale* transaction data (secondary outcome) and *participant loyalty card purchasing* data (primary outcome) from the collaborating supermarket covering three time periods: i) baseline (3 months prior to intervention implementation); ii) short-term intervention effects (0–3 months post-intervention commencement) to represent habit formation [29]; and iii) longer-term intervention effects (3–6 months post-intervention commencement) to assess sustained changes. To understand intervention effects on household members’ diets (secondary outcomes), interview-administered telephone questionnaires obtained information about participants’ and their child’s diet aged 2–6 years (where applicable) at three time points: baseline, and 3 and 6 months following intervention implementation. If a participant had more than one child aged 2–6 years, she was asked to answer questions about her eldest child in that age group. Frequency of household fruit and vegetable waste (secondary outcome) were also collected at these time points.

Store sales of fresh fruit and vegetables were provided as numbers of items for each product sold in each study week. Participant purchasing data covering the same categories were provided as the number of items for each product purchased at each store visit during the study period. Purchasing data were aggregated to present data as items per household per week. A week of sales and purchasing data were removed from the analysis if the store was closed for the full or part of the week. The time period of store closure for structural changes to the instore environment varied across intervention stores according to the size of store and extent of in-store changes. Across all intervention stores, closures affected one to four weeks of sales and purchasing data; these data were removed from the analysis for both intervention and control groups. Store sales and individual purchasing datasets consisted of 11–14 weeks before the intervention and 24–28 weeks afterwards.

Measures of women’s and children’s dietary quality were assessed using published, validated tools [30,31]. Participants were asked how often in the previous month they (or their child) consumed each of the 20 Food Frequency Questionnaire (FFQ) foods. A dietary quality score for each woman or child was calculated by multiplying their standardised reported frequency of consumption of each FFQ food by corresponding weightings derived from published tools and summing the results. Dietary quality scores were standardised (mean = 0, SD = 1). Higher scores represent better dietary quality characterised by higher intakes of vegetables, fruit, water and wholegrain bread and lower intakes of white bread, processed meats, chips, crisps and sugar. Household waste data were collected by asking participants ‘over the past month how often has your household thrown away (as a result of food spoiling or serving/cooking too much) fruit’. The same question was asked separately for vegetables. Response categories were the same as those used for the FFQs.

### 2.5. Statistical analysis

Descriptive variables are given as percentage (frequency) for categorical variables and median (interquartile range) for non-normally distributed continuous variables. Differences between participants that shopped at intervention and control stores were tested using chi-squared tests for categorical variables and Mann-Whitney rank sum tests for non-normally distributed continuous variables.

Store sales data were analysed using an Interrupted Time Series [32] (full details S2 Box). Weekly sales per store were transformed to normal distributions using Fisher–Yates transformations [33] to protect commercially sensitive sales figures (standard deviations (SDs)). The time series models were fitted separately to account for store pairing followed by random effects meta-analysis [34,35] to synthesise differences between pairs of stores at the time of intervention, and 3 and 6 months post-intervention. This method enabled: i) retention of the store pairing design, ii) comparisons between pairs and iii) overall statements of study effect size and precision [36]. Results were interpreted on the original items sold per week scale by calculating the equivalent change on the original scale to the change from the median on the Fisher–Yates transformed scale. The collaborating supermarket chain sells only packaged fruit/vegetables (not singly), with each item averaging 5 portions (~400 g). This information informed conversions from items to portions. Full evaluation of the implementation of the intervention and differences between intervention and control stores in the positioning and availability of fruit and vegetables has been published elsewhere [37]. In brief, the intervention was implemented with close adherence to the study protocol but noted some variation in the distance the produce section moved forward and the overall increase in fruit and vegetable availability in intervention stores. Hence, process evaluation results identified the need for additional analyses including stratifying by: i) distance fresh fruit and vegetables moved forwards in the store (>14 m versus <14 m), ii) post-intervention position of fresh fruit and vegetables (first/last half of first aisle), and iii) post-intervention availability of fresh fruit and vegetables (≥73/<73 Stock Keeping Units (SKUs)) [37]. These analyses involved running random effects meta-analyses on the results of the Interrupted Time Series models, described above, but with intervention stores grouped by each position/availability stratification variable, and their paired control stores.

For household purchasing data time series analysis was not possible because data were right-hand skewed (i.e., 67% of women’s weekly purchases had no fresh fruit or vegetables). Three-level multilevel models were also tested but did not fit the data but returned a singular fit, likely due to smaller than anticipated participant numbers in each store. Singular fit arose as the store variance component was estimated as effectively zero, suggesting minimal clustering at the store level. Outcome data were dichotomised to indicate whether each week resulted in any study food purchases. Two-level multilevel models, with weekly purchasing nested within women and pooling data across stores, were implemented using a difference-in-difference approach [38] (full details S2 Box). Women’s data were analysed according to their recruitment store to conform with intention-to-treat analysis. Intervention effects according to woman’s educational level (up to 16 years of age or beyond 16 years of age) were analysed according to the study protocol (secondary outcome). Process evaluation results identified the need for the same additional analyses as the store sales data, plus study supermarket as main food shop [37]. These analyses involved running models in separate stratification groups defined by education/position/availability. To determine the consistency of the effect of the intervention on household purchasing with analyses accounting for clustering at store level, two-level multilevel models were fitted in 17 store pairs individually (the model would not run in all 18 pairs because of data sparsity in one store pair), followed by random effects meta-analysis [30,31] to synthesise differences between pairs of stores. This approach could not be used for stratified analyses because a singular fit was returned.

Intervention effects on changes in dietary quality from baseline to 3 and 6 months post-intervention were assessed using linear regression models. Diet was the outcome and intervention group and diet at baseline were predictors. A second set of regression models included confounding variables (age, money spent on groceries, number of children in the household and woman’s education) determined before analyses using directed acyclic graphs (DAG) (S1 and S2 Figs) [39]. Planned multilevel linear regression models (with women clustered within stores) returned a singular fit, likely for the same reason the household purchasing data did not fit. Regression models were fitted in each pair of stores were therefore fitted separately and random effects meta-analysis [34,35] used to synthesise results. Intervention effects on household fruit and vegetable waste per week was assessed using the same approach (S3 Fig).

Analyses were performed in Stata 14 [40], except the time series models which were fitted in R using packages nlme [41,42] and msm [43]. Consistent with current statistical thinking [44] our interpretations focus on effect sizes, and their precision, rather than emphasising statistical significance relative to any boundary.

## 3. Results

### 3.1. Participant characteristics

A total of 865 women were assessed for eligibility, of which 580 were recruited; 300 were from control stores and 280 from intervention stores (Fig 1). Most women provided purchasing data (*n* = 475, 248 from control stores and 227 from intervention stores which provides 85% power at 5% significance level (2-sided) (S1 Box)) and 360 women provided information about their dietary and household fruit and vegetable waste patterns (190 from control stores and 170 from intervention stores). Of these 360 women, 250 reported living with children (aged <18 years) and 127 provided data about their child aged 2–6 years. Attrition rates for diet and waste data were 13% at 3-month and 19% at 6-month follow-ups. There were slight differences in participant characteristics at baseline between participants that shopped at intervention and control stores, with intervention women less likely to identify as being of white ethnicity and more likely to live in more deprived neighbourhoods (Table 1). More than half of the sample were aged 31–45 years, 72% identified as being white British, 40% had low educational attainment (no qualifications beyond age 16 years) and 63% were in paid employment. Almost a third reported that the study supermarket chain was where they purchased most of their groceries (31%).

**Table 1:** Baseline characteristics of all participants (*n* = 580).

Characteristic	Total (*n* = 580)	Control (*n* = 300)	Intervention (*n* = 280)	*P*-value
Age group, % (*n*)				0.45
18–30 years	16% (90)	17% (51)	14% (39)	
31–45 years	51% (298)	50% (151)	53% (147)	
46–60 years	33% (192)	33% (98)	34% (94)	
White ethnicity, % (*n*)	72% (417)	80% (239)	64% (178)	<0.001
Married/civil partnership/cohabiting, % (*n*)	61% (354)	66% (197)	56% (157)	0.05
Low education (no qualifications beyond age 16), % (*n*)	40% (224)	40% (117)	39% (107)	0.36
Most deprived half of neighbourhood deprivation (IMD), % (*n*)	69% (400)	61% (183)	78% (217)	<0.001
Paid employment, % (*n*)	63% (361)	66% (193)	60% (168)	0.16
Main supermarket used is study supermarket, % (*n*)	31% (177)	29% (85)	33% (92)	0.25
Pounds (£) spent on food per week, median (IQR)	70 (50, 100)	70 (50, 100)	70 (50, 100)	0.74
Number of children in the house, median (IQR)	1 (0, 2)	1 (0, 2)	1 (0, 2)	0.30

### 3.2. Store sales (secondary outcome)

**Fig 2:**
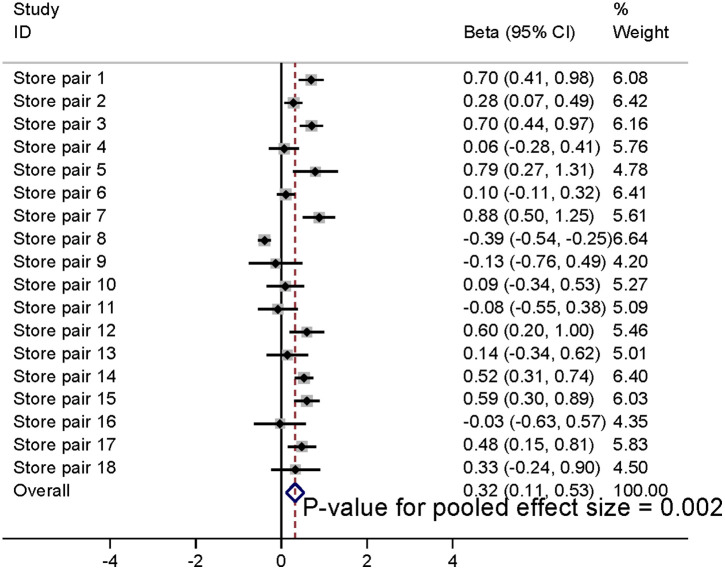
Meta-analysis of increase in store sales of fresh fruit and vegetables (SDs) in intervention stores compared to that predicted by model counterfactuals at intervention implementation.

**Fig 3:**
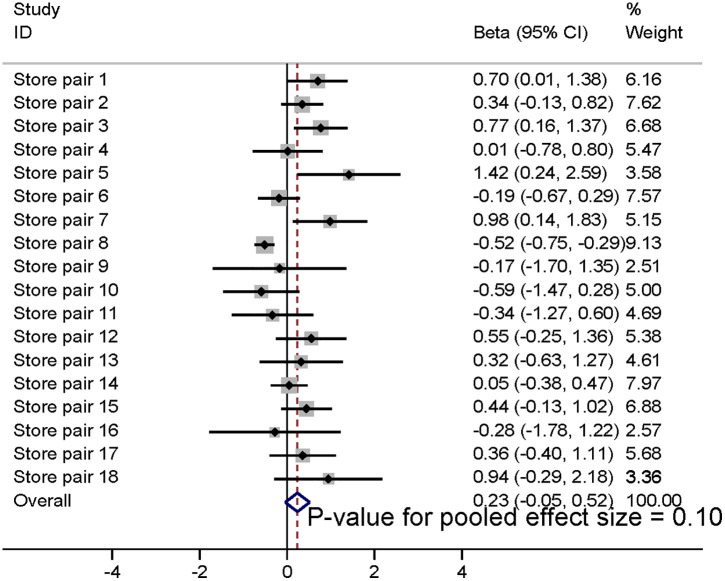
Meta-analysis of increase in store sales of fresh fruit and vegetables (SDs) in intervention stores compared to that predicted by model counterfactuals at 3 months post-intervention implementation.

**Fig 4:**
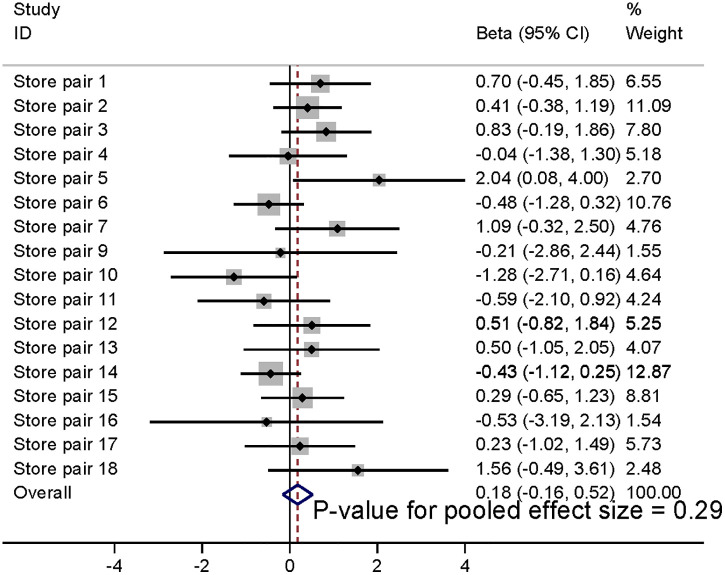
Meta-analysis of increase in store sales of fresh fruit and vegetables (SDs) in intervention stores compared to that predicted by model counterfactuals at 6 months post-intervention implementation.

The synthesised results using meta-analysis are shown in Figs 2–4, illustrating results at baseline, and 3 and 6 months post-intervention implementation, respectively. Sales of fresh fruit and vegetables were 0.32 SDs (95% CI 0.11, 0.5; *p* = 0.002) greater in the intervention stores than would be predicted by the model counterfactuals at the time of intervention; the equivalent differences were 0.23 SDs (95% CI −0.05, 0.52; *p* = 0.10) at 3 months post-intervention and 0.18 SDs (95% CI −0.16, 0.52; *p* = 0.29) at 6 months post-intervention (secondary outcome). These changes are approximately equivalent to 2,525 (95% CI 775, 4,115), 1940 (95% CI 380, 3,950) and 1,450 (95% CI −945, 3,950) extra fruit and vegetable portions per store, per week at intervention, and 3 and 6 months post-intervention implementation, respectively.

There were somewhat greater increases in sales of fresh fruit and vegetables in the intervention stores where the produce section moved forward more than 14 m (Table 2; difference = 0.48 SDs (95% CI 0.30, 0.67; *p* < 0.001) at intervention; difference = 0.40 SDs (95% CI 0.12, 0.68; *p* = 0.005) at 3 months post-intervention; difference = 0.30 SDs (95% CI −0.13, 0.74; *p* = 0.17) at 6 months post-intervention) and lower to no increase in stores where the fruit and vegetables moved fewer than 14 m further forward (difference = 0.15 SDs (95% CI −0.16, 0.45; *p* = 0.35) at intervention; difference = 0.01 SDs (95% CI −0.38, 0.40; *p* = 0.97) at 3 months post-intervention; difference = −0.01 SDs (95% CI −0.57, 0.56; *p* = 0.98) at 6 months post-intervention); however confidence intervals were wide and overlapped for stores where produce moved forward more than 14 m compared to less than 14 m. The changes in stores where fruit and vegetables moved forward more than 14 m are approximately equivalent to 3,645 (95% CI 2,350, 5,305), 3,115 (95% CI 960, 5,350) and 2,350 (95%CI 720, 6,045) greater fruit and vegetable portions per store, per week at intervention and 3 and 6 months, respectively. These findings were not replicated for intervention dose identified as being in the first, compared to the last half of the first aisle (Table 2; first half of aisle difference = 0.41 SDs (95% CI 0.25, 0.57; *p* < 0.001) at intervention; difference = 0.27 SDs (95% CI 0.02, 0.53; *p* = 0.04) at 3 months post-intervention; difference = 0.12 SDs (95% CI −0.27, 0.52; *p* = 0.53) at 6 months post-intervention compared to last half of first aisle difference = 0.09 SDs (95% CI −0.37, 0.56; *p* = 0.69) at intervention; difference = 0.15 SDs (95% CI −0.52, 0.81; *p* = 0.67) at 3 months post-intervention; difference = 0.61 SDs (95% CI −0.24, 1.47; *p* = 0.16) at 6 months post-intervention). Similarly, no notable differences in effect size were observed between stores that sold ≥73 fresh fruit and vegetables SKUs post-intervention or those which sold <73 SKUs (S1 Table; ≥ 73 SKU difference = 0.38 SDs (95% CI 0.19, 0.57; *p* < 0.001) at intervention; difference = 0.23 SDs (95% CI −0.01, 0.48; *p* = 0.06) at 3 months post-intervention; difference = 0.02 SDs (95% CI −0.39, 0.42; *p* = 0.94) at 6 months post-intervention compared to <73 SKU difference = 0.30 SDs (95% CI −0.01, 0.62; *p* = 0.06) at intervention; difference = 0.24 SDs (95% CI −0.20, 0.68; *p* = 0.28) at 3 months post-intervention; difference = 0.31 SDs (95% CI −0.24, 0.86; *p* = 0.27) at 6 months post-intervention).

**Table 2:** Increase in store sales of fresh fruit and vegetables (SDs) in intervention stores compared to that predicted by model counterfactuals at intervention, and 3- and 6-month follow-up post-intervention by dose (positioning).

Store location	Overall effect size SD (95% CI)	*P*-value	Number of stores
**All stores** ^a^			
At intervention	0.32 (0.11, 0.53)	0.002	36
12 weeks post-intervention	0.23 (−0.05, 0.52)	0.10	36
24 weeks post-intervention	0.18 (−0.16, 0.52)	0.29	36
**Fruit and vegetables moved ≥14m forwards** ^b^			
At intervention	0.48 (0.30, 0.67)	<0.001	18
12 weeks post-intervention	0.40 (0.12, 0.68)	0.005	18
24 weeks post-intervention	0.30 (−0.13, 0.74)	0.17	18
**Fruit and vegetables moved <14m forwards**			
At intervention	0.15 (−0.16, 0.45)	0.35	18
12 weeks post-intervention	0.01 (−0.38, 0.40)	0.97	18
24 weeks post-intervention	−0.01 (−0.57, 0.56)	0.98	18
**Fruit and vegetables to first half of first aisle** ^b^			
At intervention	0.41 (0.25, 0.57)	<0.001	26
12 weeks post-intervention	0.27 (0.02, 0.53)	0.04	26
24 weeks post-intervention	0.12 (−0.27, 0.52)	0.53	26
**Fruit and vegetables to last half of first aisle**			
At intervention	0.09 (−0.37, 0.56)	0.69	10
12 weeks post-intervention	0.15 (−0.52, 0.81)	0.67	10
24 weeks post-intervention	0.61 (−0.24, 1.47)	0.16	10

^a^These analyses were pre-defined in the study protocol [21].

^b^These analyses were defined from the process evaluation findings of intervention implementation [37] and offer potentially important policy relevant information.

### 3.3. Participant loyalty card purchasing data (primary outcome)

Of the 475 participants with purchasing data, there were 5,077 store visits according to their loyalty cards over the study period, 2,704 from control store participants and 2,373 from intervention store participants. Of the 5,077 visits, 1,724 (955 control women visits, 769 intervention women visits) were not at stores where women had been recruited and, while 72 of these visits were to another study store, only three visits were to stores in the opposite study arm.

**Fig 5:**
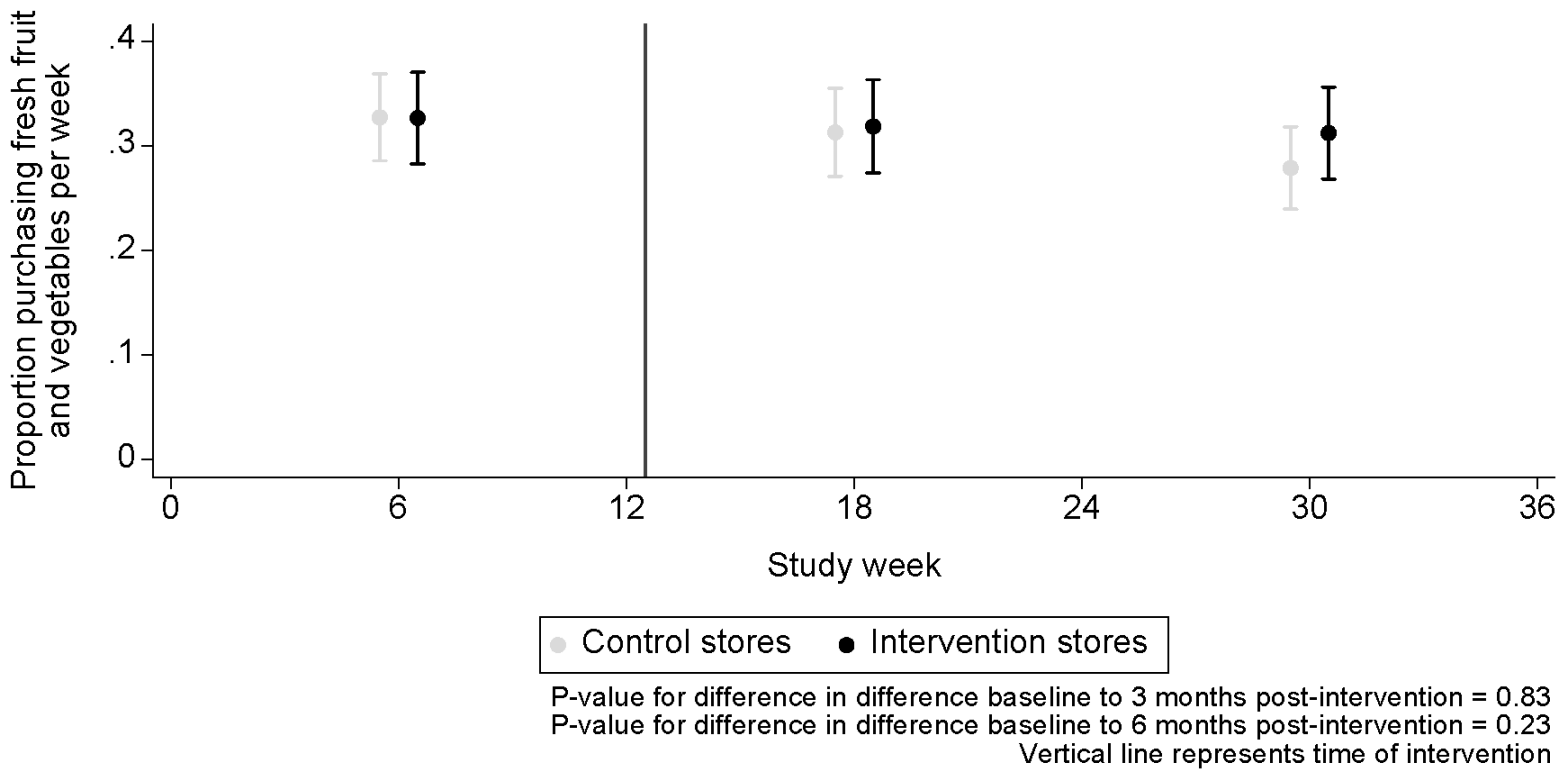
Modelled proportion of women purchasing food items in intervention and control stores.

Modelled proportions of women’s fruit and vegetable purchases show an overall decline in purchasing over time (Fig 5). The difference in the proportion of participants who purchased fruit and vegetables in intervention compared to control stores was very similar at baseline (−0.1% (95%CI −6.1%, 6.0%)) but was 0.6% (95%CI −5.6%, 6.7%) at 3 months and 3.3% (95%CI −2.5%, 9.2%) at 6 months after exposure to the intervention (Table 3) (primary outcome). In analyses accounting for clustering at store level in 17 store pairs (S4 Fig and S3 Table) consistent findings were observed to the two-level model findings in all 18 store pairs, increasing confidence in the main results.

**Table 3:** Effect of intervention on proportion of households purchasing fresh fruit and vegetables at baseline, 3- and 6-month follow-up post-intervention.

	Intervention—Control		Number of stores	Number of women	Number of visits	*P*-value for difference in difference	*P*-value for interaction
	Difference	(95% CI)					
**All participants** ^a^							
Baseline	−0.1%	(−6.1%, 6.0%)					
3 months	0.6%	(−5.6%, 6.7%)	36	475	4,793	0.83	
6 months	3.3%	(−2.5%, 9.2%)				0.23	
**Moved ≥14 m forwards** ^b^							
Baseline	0.5%	(−7.4%, 8.4%)					
3 months	2.5%	(−5.5%, 10.6%)	18	242	2,462	0.61	
6 months	5.5%	(−2.3%, 13.3%)				0.20	
**Moved <14 m forwards**							
Baseline	−1.0%	(−10.1%, 8.1%)					
3 months	−2.1%	(−11.4%, 7.1%)	18	233	2,331	0.80	0.44
6 months	0.7%	(−8.1%, 9.5%)				0.69	0.50
**Study store is main store** ^b^							
Baseline	−5.3%	(−17.0%, 6.4%)					
3 months	−1.7%	(−13.6%, 10.2%)	33	138	1,755	0.49	
6 months	4.0%	(−7.4%, 15.4%)				0.07	
**Study store is not main store**							
Baseline	1.8%	(−4.9%, 8.4%)					
3 months	0.9%	(−5.9%, 7.6%)	36	337	3,038	0.82	0.49
6 months	2.1%	(−4.5%, 8.7%)				0.91	0.18
**Educated up to age 16 years** ^a^							
Baseline	−6.2%	(−15.8%, 3.4%)					
3 months	−6.9%	(−17.0%, 3.2%)	36	180	1863	0.94	
6 months	1.5%	(−8.2%, 11.3%)				0.09	
**Educated over 16 years of age**							
Baseline	3.9%	(−3.9%, 11.7%)					
3 months	4.8%	(−3.1%, 12.7%)	35	283	2,812	0.79	0.32
6 months	5.2%	(−2.3%, 12.8%)				0.66	0.19

^a^These analyses were pre-defined in the study protocol [21].

^b^These analyses were defined from the process evaluation findings of intervention implementation [37] and offer potentially important policy relevant information.

Results for stratification by highest educational qualification, as a marker of socioeconomic status, showed a beneficial increase in the proportion purchasing fruit and vegetables amongst participants who shopped at intervention stores only after 6 months when analyses were restricted to those with fewer educational qualifications, although tests for interaction were not statistically significant by level of education (Table 3; educated up to age 16 years difference = −6.2% (95% CI −15.8%, 3.4%) at intervention; difference = −6.9% (95% CI −17.0%, 3.2%; *p* = 0.94) at 3 months post-intervention; difference = 1.5% (95% CI −8.2%, 11.3%; *p* = 0.09) at 6 months post-intervention compared to educated over 16 years of age difference = 3.9% (95% CI −3.9%, 11.7%) at intervention; difference = 4.8% (95% CI −3.1%, 12.7%; *p* = 0.79) at 3 months post-intervention; difference = 5.2% (95% CI −2.3%, 12.8%; *p* = 0.66) at 6 months post-intervention) (secondary outcome). Among participants whose main supermarket was the study store, the proportion purchasing fruit and vegetables was greater amongst those shopping at intervention stores than control stores only after 6 months of intervention exposure, although again tests for interaction did not show robust differences (Table 3; study store is main store interaction *p = 0.49* and difference = −1.7% (95% CI −13.6%, 10.2%; *p* = 0.49) at 3 months; interaction *p = 0.18* and difference = 4.0% (95% CI −7.4%, 15.4%; *p* = 0.07) at 6 months compared to study store is not main store difference = 0.9% (95% CI −5.9%, 7.6%; *p* = 0.82) at 3 months; difference = 2.1% (95% CI −4.5%, 8.7%; *p* = 0.91) at 6 months). When stratifying by intervention dose using distance produce section moved, the difference in proportion of fruit and vegetables purchasing between participants shopping at intervention verses control stores showed some increases from baseline to 3 (difference = 2.5% (95% CI −5.5%, 10.6%; *p* = 0.61) and 6 months (difference = 5.5% (95% CI −2.3%, 13.3%; *p* = 0.20) among those who shopped at stores where the produce section moved ≥14 m. The effect sizes were greater than those observed among intervention participants who shopped at intervention stores where the produce section moved <14 m (Table 3; difference = −2.1% (95% CI −11.4%, 7.1%; *p* = 0.80) at 3 months; difference = 0.7% (95% CI −8.1%, 9.5%; *p* = 0.69) at 6 months), although tests for interaction were not statistically significant. There were similarly no consistent differences in effect size according to stratification by number of SKUs (≥73 or <73) in intervention stores or for positioning of produce section in the first half or last half of the aisle in intervention stores (S2 Table).

### 3.4. Dietary variables (secondary outcomes)

Women’s diet findings (Table 4) indicate changes in dietary quality of 0.07 SDs (95% CI −0.09, 0.23) at 1 month post-intervention, −0.15 SDs (95% CI −0.33, 0.03) at 3 months post-intervention and 0.25 SDs (95% CI 0.10, 0.40) at 6 months post-intervention (secondary outcome). Equivalent changes for children’s dietary quality were 0.34 SDs (95% CI −0.30, 0.98), 0.43 SDs (95% CI −0.39, 1.25) and 0.06 SDs (95% CI −0.13, 0.24) at 1-, 3- and 6-month follow-up, respectively, with uncertainty indicated by wide confidence intervals (secondary outcome).

**Table 4:** Effect of store intervention on women’s and children’s dietary quality from baseline to 1-, 3- and 6-month follow-up post-intervention.

	Unadjusted					Adjusted^*^				
Outcome	Change in dietary quality score (SD)^**^	(95% CI)	*P*-value	*n* participants	*n* stores	Change in dietary quality score (SD)^**^	(95% CI)	*P*-value	*n* participants	*n* stores
***Women’s diet*** **Baseline to 1 month**										
Dietary quality (SDs)	0.02	(−0.11, 0.16)	0.74	327	34	0.07	(−0.09, 0.23)	0.40	298	34
**Baseline to 3 months**										
Dietary quality (SDs)	−0.04	(−0.18, 0.10)	0.57	313	36	−0.15	(−0.33, 0.03)	0.11	286	36
**Baseline to 6 months**										
Dietary quality (SDs)	0.06	(−0.07, 0.18)	0.37	290	36	0.25	(0.10, 0.40)	0.001	252	32
***Children’s diet*** **Baseline to 1 month**										
Dietary quality (SDs)	0.13	(−0.23, 0.49)	0.47	94	20	0.34	(−0.30, 0.98)	0.30	75	14
**Baseline to 3 months**										
Dietary quality (SDs)	0.16	(−0.26, 0.59)	0.45	82	18	0.43	(−0.39, 1.25)	0.31	70	14
**Baseline to 6 months**										
Dietary quality (SDs)	−0.02	(−0.25, 0.21)	0.87	73	18	0.06	(−0.13, 0.24)	0.55	49	10

* Adjusted for age, money spent on groceries, children in household, woman’s education. This results in some pairs of stores being excluded from the analyses due to insufficient numbers of participants.

** SD, standard deviation.

**Table 5:** Effect of store intervention on change in frequency of waste from baseline to 1-, 3- and 6-month follow-up post-intervention.

	Unadjusted					Adjusted^*^				
**Outcome**	**Change in food waste score** **(SD)** ^**^	**(95% CI)**	***P*-value**	***n* women**	***n* store pairs**	**Change in food waste score** **(SD)** ^**^	**(95% CI)**	***P*-value**	***n* women**	***n* store pairs**
**Baseline to 1 month**										
Fruit waste (frequency per week)	0.06	(−0.06, 0.19)	0.31	324	17	−0.01	(−0.24, 0.22)	0.93	295	17
Vegetable waste (frequency per week)	0.04	(−0.06, 0.13)	0.44	324	17	−0.02	(−0.20, 0.17)	0.87	295	17
**Baseline to 3 months**										
Fruit waste (frequency per week)	0.04	(−0.11, 0.19)	0.60	309	18	0.07	(−0.18, 0.32)	0.57	282	18
Vegetable waste (frequency per week)	0.06	(−0.04, 0.17)	0.24	309	18	−0.02	(−0.25, 0.20)	0.83	282	18
**Baseline to 6 months**										
Fruit waste (frequency per week)	−0.04	(−0.18, 0.10)	0.60	288	18	0.12	(−0.12, 0.35)	0.32	264	18
Vegetable waste (frequency per week)	0.12	(0.02, 0.23)	0.02	288	18	0.20	(0.07, 0.32)	0.002	264	18

* Adjusted for money spent on groceries, children in household, woman’s education.

** SD, standard deviation.

### 3.5. Household waste (secondary outcome)

Results for reported household waste (Table 5) revealed little change in frequency of fresh fruit being thrown away at 1- (−0.01 times per week (95% CI −0.24, 0.22)) and 3-month follow-up (0.07 times per week (95% CI −0.18, 0.32)) (secondary outcome). Similarly, there was little effect on change in frequency of fresh vegetables being thrown away at 1- (−0.02 times per week (95% CI −0.20, 0.17) and 3-month follow-up ((−0.02 times per week (95% CI −0.25, 0.20)) (secondary outcome). At 6 months post-intervention, differences in reported frequency of waste in intervention compared to control stores were 0.20 times per week (95% CI 0.07, 0.32) for vegetables and 0.12 times per week (95% CI −0.12, 0.35) for fruit.

## 4. Discussion

This trial in discount supermarkets to enhance the placement (positioning and availability) of fresh fruit and vegetables resulted in increased fresh fruit and vegetable sales at a population (store) level at the time of intervention implementation, though the size and robustness of the effect weakened over the 6-month follow-up period. These results are important given population-level declines in fruit and vegetable sales and intake in the UK over the period of the study [45,46]. Findings also showed an even greater increase on sales of fruit and vegetables in the stores where produce moved further forward, though size and robustness of these effects also weakened over time. The analyses investigating the dose of the intervention were not pre-specified but determined from investigations of implementation of the intervention published in a companion paper [37]. Among stores with the higher intervention dose approximately 3,645, 3,115 and 2,350 additional portions of fruit and vegetables were sold in each intervention store, per week at the time of intervention implementation, and 3- and 6 months later, respectively. These figures could translate to clinically meaningful improvements to population health because an increase of 50 g fruit and vegetables per day (1 portion is 80 g) has been associated with 20% reduction in all-cause mortality [47].

At the household level, the results suggest the intervention may have a small beneficial effect on fresh fruit and vegetable purchasing patterns, particularly after 6 months of intervention implementation. At a time when fruit and vegetable purchasing and intake were declining across the UK population [45,48], the results indicate a smaller reduction in fruit and vegetable purchasing among intervention compared to control families. The results also suggest that among families where the woman shopper has obtained no formal educational qualifications beyond school, those shopping in intervention stores may receive slightly greater benefit from the intervention than those with further educational qualifications, with differences in the proportion of intervention families purchasing fresh fruit and vegetables increasing after continued intervention exposure for 6 months. Similar results were observed for families using the study stores for most of their groceries (analyses not pre-specified). Women’s dietary quality improved after 6 months of exposure to the intervention, compared to women not exposed to the intervention. However, beneficial effects of the intervention were not observed for diet after 1 or 3 months of intervention exposure. Children’s dietary quality improved somewhat immediately and shortly after the intervention. But uncertainty surrounds these results and no change in dietary quality was observed after 6 months. Self-reported household waste of fruit and vegetables was not different between intervention and control families immediately and shortly after intervention exposure, however, intervention families reported higher vegetable waste after 6 months compared to baseline.

This fresh fruit and vegetable placement trial in supermarkets is novel in its assessment of the effects on household purchasing patterns using loyalty card data alongside multiple datasets to provide a thorough overview of intervention impact. Other advantages over existing research [20] includes using: a matched comparison group; robust statistical methods; and assessment of differential interventions effects by socioeconomic status and intervention dose.

The study limitation of not being able to randomise stores potentially biases the results through unmeasured confounding effects. Additionally, findings related to intervention dose were not prespecified but determined from process evaluation findings investigating intervention implementation [37]. Nonetheless, the findings provide information about intervention effectiveness in complex social contexts which is useful for policymakers. Intended three-level multilevel models [21] returned a singular fit, most likely because of smaller than predicted participant numbers in some stores due to data collection disruptions during the COVID-19 pandemic and cost-of-living crisis. The two-level multilevel models used for purchasing data analyses therefore do not account for clustering of participants within stores but rather pool the data across stores. Results are from complete case analyses which may lead to biased results and reduced statistical power, particularly when data are not missing completely at random. Interaction analyses were conducted on sub-groups increasing uncertainty in the findings [49]. The use of random-effect meta-analysis to synthesise results may have decreased the precision of our results, although the estimates should be unbiased [50]. Individual dietary and household waste results should be viewed cautiously due to difficulties recruiting over COVID-19 which resulted in smaller than predicted samples and the potential for results to be driven by chance. Store waste figures were not collected, and data from other supermarkets used by participants were not available. Store selection and intervention implementation was not within the researchers’ control and some deviations from the protocol were identified through the study’s process evaluation [37]. Under- or over-estimation of intervention effects may be possible; however, stratified analyses attempted to address known variations in intervention implementation.

Previous intervention studies which repositioned fresh fruit and vegetables to store entrances or other prominent locations in food stores found similar effects to our study [51–53]. The intervention dose (i.e., distance produce section moved and study store being main source of groceries) moderately boosted sales of fruit and vegetables at a population level and somewhat protected against declining fruit and vegetable purchasing at the household level. Previous studies of lower intensity fruit and vegetable positioning interventions, like co-locating produce alongside unhealthy foods or having smaller displays, have shown no intervention effect or only very small intervention effects [51,53,54].

Our study highlights the worrying decline in fresh fruit and vegetable sales from retail outlets in the UK. From 2018 to 2022, British families experienced disruptions to produce availability and price rises caused by COVID-19, Brexit, climatic events, and war in Europe. Subsequently, UK household purchasing of fruit dropped by 7.2% and vegetable purchasing dropped by 5.3% [45]. Households are purchasing, on average, fewer than four portions of fruit and vegetables per day for the entire family. With approximately 2.4 residents in UK households, it is not surprising only 33% of adults meet the recommended 5-a-day [55]. Even more worrying, national dietary data showed the average fruit and vegetable intake among adults in England has declined 1 portion a day from 2019 to 2023 [46]. Our study shows a slight reduction in intervention effect size on store-level sales 6 months post-intervention implementation which mirrors population trends. Even small increases in fruit and vegetable consumption (0.3–1.0 portion/day) can reduce an individual’s risk of coronary heart disease by 4% and stroke by 5% [56,57]. Our study results suggest that this intervention could potentially offer a meaningful population health benefit despite contextual disruptions.

At the household level the intervention provided somewhat protective effects against these national trends, with more convincing results at the household and individual levels after 6 months of continued intervention exposure and among families relying on the study store for most of their groceries and experiencing lower socioeconomic position (determined by women’s educational attainment). Evidence from the US shows similar findings for families receiving governmental financial support to buy nutritious foods for their young children, with higher use after five months of continued exposure to produce sections near their store’s entrance [52]. The broader literature on changing health-related practices indicates that new habits take more than 12 weeks to form and strengthen with time [58], particularly if contexts or environments support those new practices [59,60]. Observational evidence shows UK discount and small supermarkets who attract families with lower incomes do not provide this contextual support, frequently not positioning fruit and vegetable sections near store entrances [5,15]. Positioning produce sections in this prominent in-store location across all supermarkets could provide small protective benefits for those most at risk of low fruit and vegetable intake.

Our study, combined with the broader evidence base in this field, provide contextually relevant considerations for food policy. The store level findings suggest the government could consider expansion of the UK Food (Promotions and Placement) regulations in England to require the positioning of fresh produce sections near store entrances in all large food stores (>2,000 square feet) to boost fruit and vegetable sales and improve population diet. The international literature indicates that concurrently positioning healthy foods in prominent locations and repositioning unhealthy foods to less prominent locations is more beneficial for diet-related outcomes than co-locating unhealthy and healthy items in prominent locations or implementing only one positioning strategy [11,61]. While the UK Ofcom 2004/2005 Nutrient Profile Model [62] used to define unhealthy foods for the Food (Promotions and Placement) regulations has prompted reformulation of savoury snacks and pizzas sold in retail outlets [63], it does not adhere to current UK dietary guidelines and still allows prominent positioning of less healthy, high-sugar foods. Policy refinement to also require the positioning of healthy foods, like fruit and vegetables, at prominent in-store locations would help maximise the regulations’ population health impact [64]. By illustration, findings from the pilot phase of our study, which concurrently increased the placement (availability and positioning) of produce and removed unhealthy items from checkouts and aisle ends opposite (replaced with non-food items), showed an increase of approximately 9,820 additional fruit and vegetable portions per store per week after 6 months [24]. Comparatively, the current full-scale study, which only improved the placement of produce, revealed an increase of approximately 1,450 additional portions per store per week after 6 months. These findings demonstrate the added benefit of combining placement strategies for both healthy and unhealthy products.

This study provides a comprehensive assessment of a fruit and vegetable placement intervention where positioning and availability were enhanced in discount supermarkets. Findings show increased fresh fruit and vegetable sales at the population level at the time of intervention, although the size and robustness reduced over time. Findings also suggest there may be small protective benefit from the intervention for household fruit and vegetable purchasing and individual dietary quality after continued intervention exposure because it buffered against widescale reductions in fruit and vegetable purchasing and intake caused by COVID-19 and cost-of-living challenges from 2019 to 2023, particularly among families relying on intervention stores for their groceries and experiencing greater socioeconomic disadvantage. This study alongside the broader evidence base in this field provides evidence for governments to consider implementing regulations to improve retailers’ in-store food marketing practices.

## Supporting information

S1 Fig Directed Acyclic Graph for women’s dietary quality. (DOCX)

S2 Fig Directed Acyclic Graph for children’s dietary quality. (DOCX)

S3 Fig Directed Acyclic Graph for household fruit and vegetable waste. (DOCX)

S4 Fig Modelled proportion of women purchasing food items in intervention and control stores amongst 17 store pairs where results could be fitted using two level multilevel models in each store pair and combined using meta-analysis. (DOCX)

S1 Box Sample size calculation. (DOCX)

S2 Box Interrupted Time Series analysis—store sales data and Difference in Difference analyses—household purchasing data. (DOCX)

S1 Table Increase in store sales of fresh fruit and vegetables (SDs) in intervention stores compared to that predicted by model counterfactuals at intervention, and 3- and 6-month follow-up post-intervention by dose (availability). (DOCX)

S2 Table Effect of intervention on change in proportion of fresh fruit and vegetable purchasing from baseline to 3- and 6-month follow-up post-intervention stratified by dose (position and availability). (DOCX)

S3 Table Effect of intervention on proportion of households purchasing fresh fruit and vegetables at baseline, 3- and 6 month follow-up post-intervention amongst 17 store pairs where results could be fitted using two level multilevel models in each store pair and combined using. (DOCX)

S1 CONSORT Checklist CONSORT 2010 Statement. Updated guidelines for reporting parallel group randomised trials. 2010 Schulz and colleagues. This is an Open Access article distributed under the terms of the Creative Commons Attribution License (http://creativecommons.org/licenses/by/2.0), which permits unrestricted use, distribution, and reproduction in any medium, provided the original work is properly cited.
(DOC)

S1 CONSERVE Checklist CONSERVE Checklists. (DOCX)

## Abbreviations

IMD: Index of Multiple Deprivation
FFQ: Food Frequency Questionnaire
SDs: Standard Deviations
SKUs: Stock Keeping Units
