# Supplementary figures and images for: Impact of supermarket fruit and vegetable placement on store sales, customer purchasing, diet and household waste: A prospective matched-controlled cluster trial
Source: PLoS Med. 2026 Mar 31;23(3):e1004575. doi: 10.1371/journal.pmed.1004575 (PMC13038019; doi:10.1371/journal.pmed.1004575)

**S1 Fig: Directed Acyclic Graph for women’s dietary quality**

**
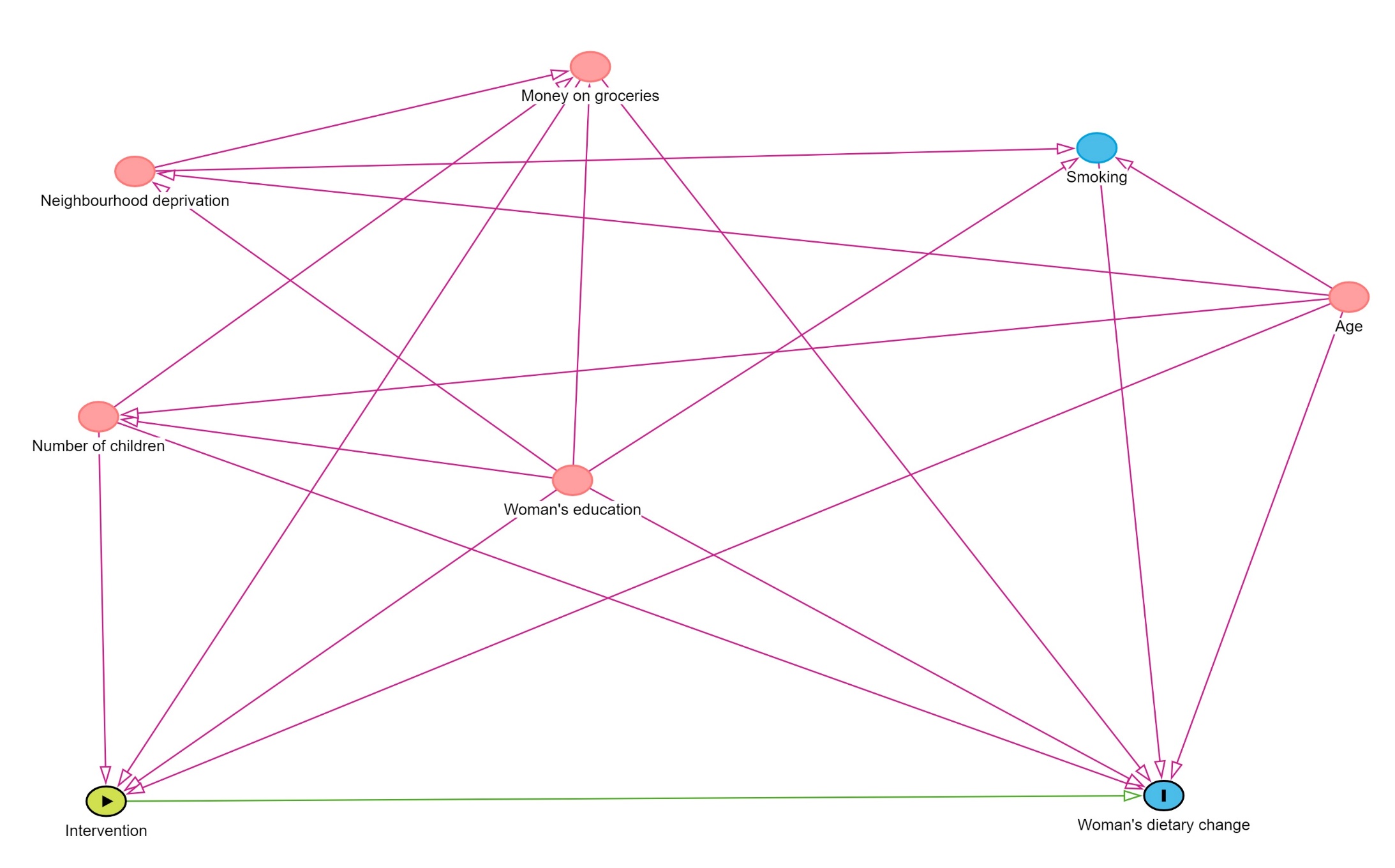
**

Supplement: S1 Fig — (DOCX) [file pmed.1004575.s001.docx]

**S2 Fig: Directed Acyclic Graph for children’s dietary quality**

**
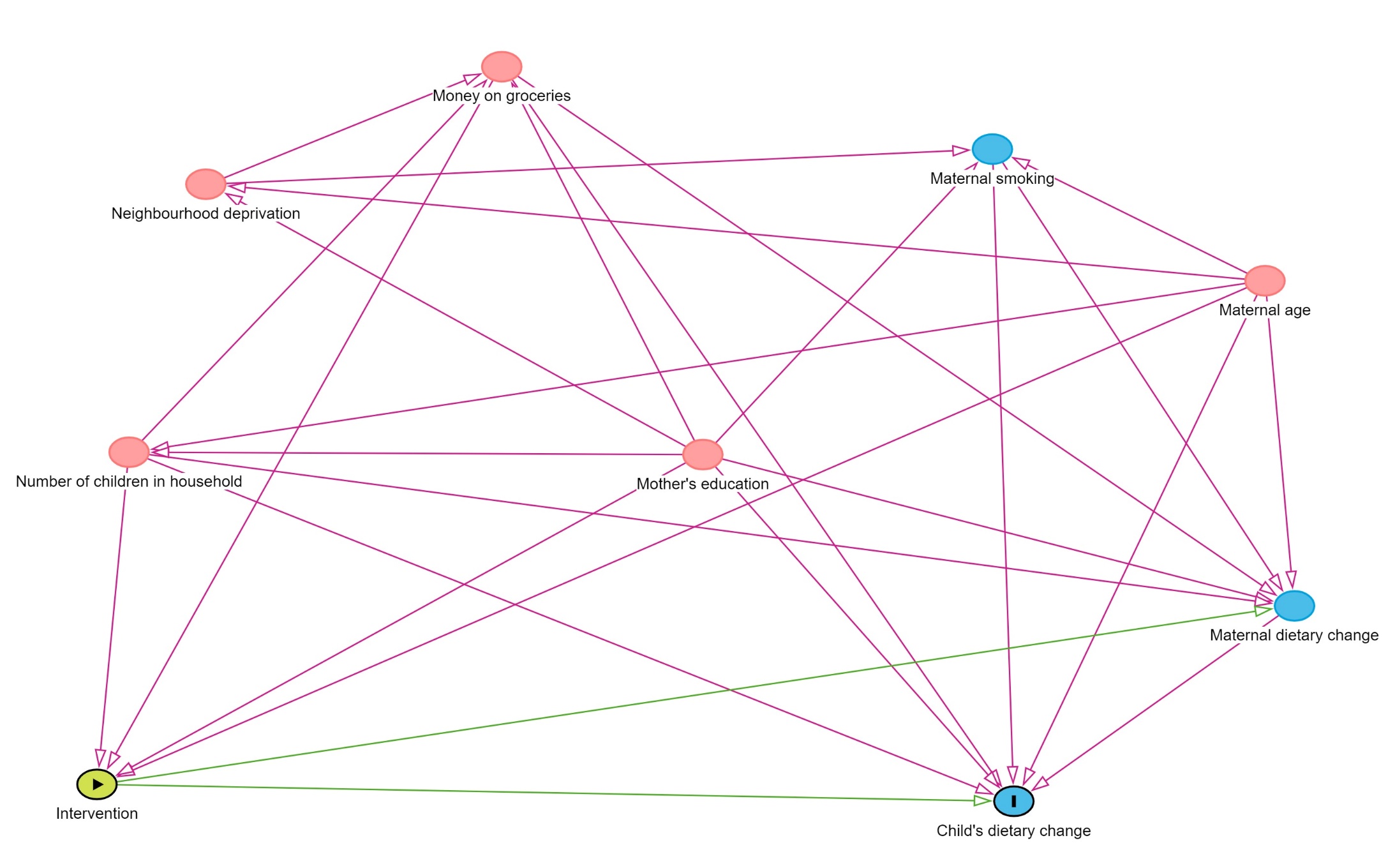
**

Supplement: S2 Fig — (DOCX) [file pmed.1004575.s002.docx]

**S3 Fig: Directed Acyclic Graph for household fruit and vegetable waste**

**
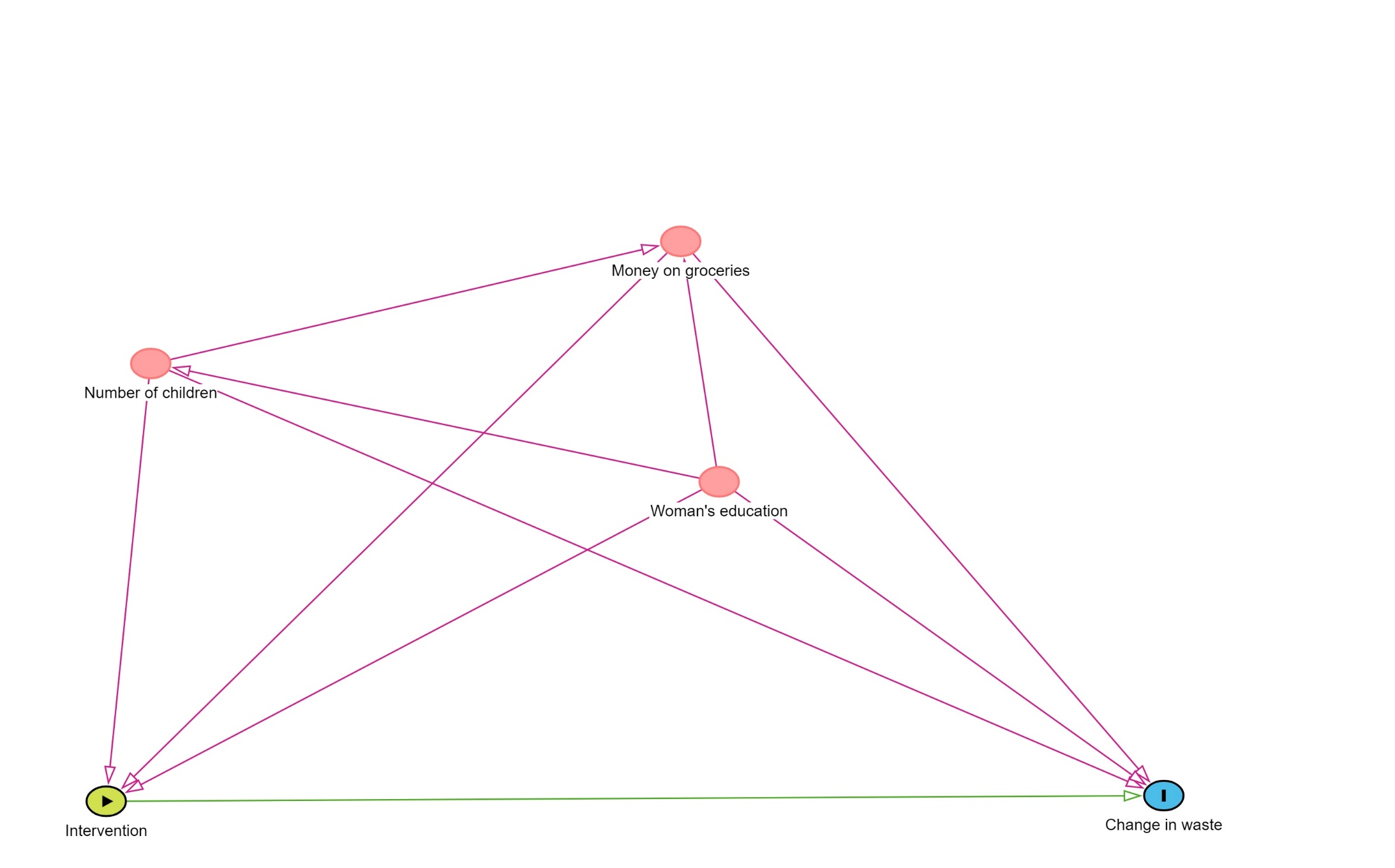
**

Supplement: S3 Fig — (DOCX) [file pmed.1004575.s003.docx]
