# Supplementary material for: Impact of supermarket fruit and vegetable placement on store sales, customer purchasing, diet and household waste: A prospective matched-controlled cluster trial
Source: PLoS Med. 2026 Mar 31;23(3):e1004575. doi: 10.1371/journal.pmed.1004575 (PMC13038019; doi:10.1371/journal.pmed.1004575)
Supplement: S4 Fig — (DOCX) [file pmed.1004575.s004.docx]

**S4 Fig:** **Modelled proportion of women purchasing food items in intervention and control stores amongst 17 store pairs where results could be fitted using two level multilevel models in each store pair and combined using meta-analysis**
