# Supplementary material for: Impact of supermarket fruit and vegetable placement on store sales, customer purchasing, diet and household waste: A prospective matched-controlled cluster trial
Source: PLoS Med. 2026 Mar 31;23(3):e1004575. doi: 10.1371/journal.pmed.1004575 (PMC13038019; doi:10.1371/journal.pmed.1004575)
Supplement: S1 Box — (DOCX) [file pmed.1004575.s005.docx]

**Supplementary Box 1**

**Sample size calculation**

The study’s sample size calculations were revised post-hoc because of COVID-19 related disruptions to participant recruitment. The sixth wave of recruitment undertaken to boost participant numbers resulted in this study being powered to detect differences in the primary outcome (fresh fruit and vegetable purchasing) between women in the intervention and control groups during the 3-6 months post-intervention. Average fruit and vegetable purchases per week are not normally distributed, thus the sample size calculation is based on changes in average fresh fruit and vegetable purchases per week from the baseline period to the post-intervention period, which are approximately normally distributed. It was not practical to calculate a rho from the pilot study due to the small number of clusters. We used data from our previous research on women in Hampshire who were the same age-range as the proposed participants of this study and considered the supermarkets at which the women shopped as clusters to estimate a rho of 0.1 as our intraclass correlation coefficient. We aimed to detect a difference of 0.3 items (1.5 portions) per week. Assuming a standard deviation of 0.7 items (3.5 portions) per week as seen in the pilot data, 18 stores in each arm and 13 women per store (totaling 468 women) provides 85% power at a 5% significance level (2-sided).
