# Supplementary material for: Impact of supermarket fruit and vegetable placement on store sales, customer purchasing, diet and household waste: A prospective matched-controlled cluster trial
Source: PLoS Med. 2026 Mar 31;23(3):e1004575. doi: 10.1371/journal.pmed.1004575 (PMC13038019; doi:10.1371/journal.pmed.1004575)
Supplement: S2 Box — (DOCX) [file pmed.1004575.s006.docx]

**S2 Box**

**Interrupted Time Series analysis – store sales data**

Time series models were fitted with terms for study week (linear term, weeks from baseline), intervention, level (an indicator of the post-intervention period), trend (study week in the post-intervention period), and interactions between intervention and study week, intervention and level and intervention and trend. Additional terms were included in the models for Christmas weeks, the week at the end of lockdown in June 2020 and the week before the intervention to improve model fit. By including the variable ‘level’, the model tested for a step change at the time of the intervention. The time series models were fitted separately in each pair of stores to account for store pairing. The P-value for the interaction between intervention and level indicates the significance of the impact of the intervention on store sales at the time of the intervention. Effect sizes were calculated at the 3- and 6-months post-intervention time points. A counterfactual line is included on the Interrupted Time Series graphs, indicating the trends in sales that would have been expected had the intervention not occurred. Confidence intervals at 3- and 6-months post-intervention were calculated using the delta method[1].

**Difference in difference analysis – household purchasing data**

A difference-in-difference approach was used [2] where each logistic regression model included fixed effects for intervention group, time period and the interaction between intervention group and time period. Time period was coded as two dummy variables indicating the 0-3- and 3-6-month periods post-intervention. The interaction terms tested the difference in purchasing during the intervention compared to the pre-intervention period between intervention and control stores. Random effects were included for women, to account for the multilevel structure of the data, with weeks clustered within women.
