# Supplementary material for: Impact of supermarket fruit and vegetable placement on store sales, customer purchasing, diet and household waste: A prospective matched-controlled cluster trial
Source: PLoS Med. 2026 Mar 31;23(3):e1004575. doi: 10.1371/journal.pmed.1004575 (PMC13038019; doi:10.1371/journal.pmed.1004575)
Supplement: S1 Table — (DOCX) [file pmed.1004575.s007.docx]

**S1 Table: Increase in store sales of fresh fruit and vegetables (SDs) in intervention stores compared to that predicted by model counterfactuals at intervention, and 3- and 6-months follow-up post-intervention by dose (availability)**

| **Store location** | **Overall effect size 95% CI** | **P-value** | **Number of stores** |
| --- | --- | --- | --- |
| All stores |  |  |  |
| At intervention | 0.32 (0.10, 0.53) | 0.004 | 36 |
| 12 weeks post-intervention | 0.23 (-0.05, 0.52) | 0.11 | 36 |
| 24 weeks post-intervention | 0.18 (-0.16, 0.52) | 0.29 | 36 |
| **≥ 73 SKU** |  |  |  |
| At intervention | 0.38 (0.19, 0.57) | < 0.001 | 16 |
| 12 weeks post-intervention | 0.23 (-0.01, 0.48) | 0.06 | 16 |
| 24 weeks post-intervention | 0.02 (-0.39, 0.42) | 0.94 | 16 |
| **< 73 SKU** |  |  |  |
| At intervention | 0.30 (-0.01, 0.62) | 0.06 | 20 |
| 12 weeks post-intervention | 0.24 (-0.20, 0.68) | 0.28 | 20 |
| 24 weeks post-intervention | 0.31 (-0.24, 0.86) | 0.27 | 20 |
