# Supplementary material for: Impact of supermarket fruit and vegetable placement on store sales, customer purchasing, diet and household waste: A prospective matched-controlled cluster trial
Source: PLoS Med. 2026 Mar 31;23(3):e1004575. doi: 10.1371/journal.pmed.1004575 (PMC13038019; doi:10.1371/journal.pmed.1004575)
Supplement: S2 Table — (DOCX) [file pmed.1004575.s008.docx]

**S2 Table: Effect of intervention on proportion of households purchasing fresh fruit and vegetables at baseline, 3- and 6-months follow-up post-intervention stratified by dose (position and availability)**

|  | **Intervention - Control** | | **Number of stores** | **Number of women** | **Number of visits** | **P-value for difference in** | **P-value for interaction** |
| --- | --- | --- | --- | --- | --- | --- | --- |
|  | **Difference** | **(95% CI)** |  |  |  | **difference** |  |
| **Moved first half of first aisle** |  |  |  |  |  |  |  |
| Baseline | 1.9% | (-5.6%, 9.3%) | 26 | 313 | 3295 |  |  |
| 3 months | -0.6% | (-8.0%, 6.7%) |  |  |  | 0.51 |  |
| 6 months | 5.3% | (-1.9%, 12.5%) |  |  |  | 0.33 |  |
| **Moved last half of first aisle** |  |  |  |  |  |  |  |
| Baseline | -4.3% | (-15.1%, 6.5%) | 10 | 162 | 1498 |  |  |
| 3 months | 1.8% | (-9.6%, 13.2%) |  |  |  | 0.25 | 0.20 |
| 6 months | -0.3% | (-11.1%, 10.5%) |  |  |  | 0.46 | 0.96 |
| **≥ 73 SKU** |  |  |  |  |  |  |  |
| Baseline | 2.0% | (-7.3%, 11.2%) | 16 | 216 | 2051 |  |  |
| 3 months | 0.1% | (-9.3%, 9.6%) |  |  |  | 0.69 |  |
| 6 months | 8.1% | (-1.1%, 17.3%) |  |  |  | 0.17 |  |
| **< 73 SKU** |  |  |  |  |  |  |  |
| Baseline | -1.5% | (-9.5%, 6.4%) | 20 | 259 | 2742 |  |  |
| 3 months | 0.9% | (-7.1%, 8.9%) |  |  |  | 0.54 | 0.50 |
| 6 months | -0.6% | (-8.2%, 6.9%) |  |  |  | 0.84 | 0.36 |
