# Supplementary material for: Impact of supermarket fruit and vegetable placement on store sales, customer purchasing, diet and household waste: A prospective matched-controlled cluster trial
Source: PLoS Med. 2026 Mar 31;23(3):e1004575. doi: 10.1371/journal.pmed.1004575 (PMC13038019; doi:10.1371/journal.pmed.1004575)
Supplement: S3 Table — (DOCX) [file pmed.1004575.s009.docx]

**S3 Table: Effect of intervention on proportion of households purchasing fresh fruit and vegetables at baseline, 3- and 6 months follow-up post-intervention amongst 17 store pairs where results could be fitted using two level multilevel models in each store pair and combined using meta-analysis**

|  | **Intervention - Control** | | **Number of stores** | **Number of women** | **Number of visits** | **P-value for difference in** |
| --- | --- | --- | --- | --- | --- | --- |
|  | **Difference** | **(95% CI)** |  |  |  | **difference** |
| Baseline | -0.5% | (-6.5, 5.4%) | 34 | 461 | 4701 |  |
| 3 months | 2.2% | (-3.7%, 8.1%) |  |  |  | 0.81 |
| 6 months | 2.8% | (-3.0%, 8.7%) |  |  |  | 0.34 |
