# Supplementary material for: Impact of supermarket fruit and vegetable placement on store sales, customer purchasing, diet and household waste: A prospective matched-controlled cluster trial
Source: PLoS Med. 2026 Mar 31;23(3):e1004575. doi: 10.1371/journal.pmed.1004575 (PMC13038019; doi:10.1371/journal.pmed.1004575)
Supplement: S1 CONSERVE Checklist — (DOCX) [file pmed.1004575.s011.docx]

# CONSERVE Checklists

Use CONSERVE-CONSORT for completed trial reports and CONSERVE-SPIRIT for trial protocols.

| CONSERVE-CONSORT Extension: [DATE] | | | | | |
| --- | --- | --- | --- | --- | --- |
| Item | Item Title | Description | | | Page No/ Section, paragraphs |
| I. | Extenuating Circumstances | Describe the circumstances and how they constitute extenuating circumstances. | | | Methods, paragraphs 7-8 |
| II. | Important Modifications | 1. Describe how the modifications are important modifications. | | | Methods, paragraphs 7-8 |
|  |  | 1. Describe the impacts and mitigating strategies, including their rationale and implications for the trial. | | | Methods, paragraphs 7-8, 13-17  Discussion, paragraphs 1-3 |
|  |  | 1. Provide a modification timeline. | | | Methods, paragraph 13 |
| III. | Responsible Parties | State who planned, reviewed and approved the modifications. | | | Methods, paragraph 1 |
| IV. | Interim data | If modifications were informed by trial data, describe how the interim data were used, including whether they were examined by study group, and whether the individuals reviewing the data were blinded to the treatment allocation. | | | N/A |
| CONSORT Number and Item | | For each row, if important modifications occurred check “direct impact” and/or “mitigating strategy” and describe the changes in the trial manuscript or supplement. Check “no change” for items that are unaffected in the extenuating circumstance. | | | Page No. |
|  |  | No Change | Impact* | Mitigating Strategy** |  |
| 1 | Title and abstract |  |  | x | Abstract, paragraph 2 |
| 2 | Introduction | X |  |  |  |
| 3 | Methods: Trial Design | X |  |  |  |
| 4 | Methods: Participants |  |  | x | Methods, paragraph 13 |
| 5 | Methods: Interventions | X |  |  |  |
| 6 | Methods: Outcomes | X |  |  |  |
| 7 | Methods: Sample Size |  |  | x | Supp fig 4 |
| 8-10 | Methods: Randomisation | X |  |  |  |
| 11 | Methods: Blinding | X |  |  |  |
| 12 | Methods: Statistical methods |  |  | x | Methods, paragraphs 13-17 |
| 13 | Results: Participant flow | X |  |  |  |
| 14 | Results: Recruitment | X |  |  |  |
| 15 | Results: Baseline data | X |  |  |  |
| 16 | Results: Numbers analysed | X |  |  |  |
| 17 | Results: Outcomes and estimation | X |  |  |  |
| 18 | Results: Ancillary analyses |  |  | x | Methods, paragraphs 15-17, Tables 2-5 |
| 19 | Results: Harms | X |  |  |  |
| 20 | Discussion: Limitations |  |  | X | Discussion, paragraph 4 |
| 21 | Discussion: Generalisability | X |  |  |  |
| 23 | Other information: Registration | X |  |  | Page 2 |
| 24 | Other information: Protocol |  |  | x | Methods, paragraph 1, Reference 22 |
| 25 | Other information: Funding | X |  |  | Page 2 |
| *Aspects of the trial that are directly affected or changed by the extenuating circumstance and are not under the control of investigators, sponsor or funder.  **Aspects of the trial that are modified by the study investigators, sponsor or funder to respond to the extenuating circumstance or manage the direct impacts on the trial. | | | | | |
